# Interleukin-22 Polymorphisms in *Plasmodium falciparum*-Infected Malaria Patients

**DOI:** 10.1155/2020/5193723

**Published:** 2020-02-19

**Authors:** Nada H. Aljarba, Mashael R. Al-Anazi, Mohammed I. Shafeai, Fuad H. Rudiny, Saad M. Bin Dajem, Hani Alothaid, Majid Darraj, Saad Alkahtani, Jahad Alghamdi, Mohammed N. Al-Ahdal, Ahmed A. Al-Qahtani

**Affiliations:** ^1^Biology Department, College of Science, Princess Nourah bint Abdulrahman University, Riyadh, Saudi Arabia; ^2^Department of Infection and Immunity, Research Centre, King Faisal Specialist Hospital & Research Centre, Riyadh, Saudi Arabia; ^3^Sabya General Hospital, Sabya, Saudi Arabia; ^4^Department of Biology, College of Science, King Khalid University, Abha, Saudi Arabia; ^5^Department of Basic Sciences, Faculty of Applied Medical Sciences, Al-Baha University, Al-Baha, Saudi Arabia; ^6^Department of Internal Medicine, College of Medicine, Jazan University, Jazan, Saudi Arabia; ^7^Department of Zoology, College of Science, King Saud University, Riyadh, Saudi Arabia; ^8^The Saudi Biobank, King Abdullah International Medical Research Center, King Saud bin Abdulaziz University for Health Sciences, Ministry of National Guard Health Affairs, Riyadh, Saudi Arabia; ^9^Department of Microbiology and Immunology, Alfaisal University, School of Medicine, Riyadh, Saudi Arabia

## Abstract

*Background and Objectives*. Malaria infection, caused by *Plasmodium falciparum*, is the most lethal and frequently culminates in severe clinical complications. Interleukin-22 (IL-22) has been implicated in several diseases including malaria. The objective of this study was to investigate the role of IL-22 gene polymorphisms in *P*. *falciparum* infection. *Material and Methods*. Ten single-nucleotide polymorphisms (SNPs), rs976748, rs1179246, rs2046068, rs1182844, rs2227508, rs2227513, rs2227478, rs2227481, rs2227491, and rs2227483, of *IL-22* gene were genotyped through PCR-based assays of 250 *P*. *falciparum* infection. *IL*-*22* gene promoter activity.

**Results:**

We found that the rs2227481 TT genotype (odds ratio 0.254, confidence interval = 0.097-0.663, *P*. *P*. *falciparum* infection. *P*. *P*. *P*. *P*.

**Conclusion:**

The study suggests that IL-22 polymorphisms in rs2227481 and rs2227483 could contribute to protection against *P*. *falciparum* infection. *IL*-*22* gene promoter activity.

## 1. Introduction

Malaria caused by *Plasmodium falciparum* is associated with significant morbidity and mortality in humans [1]. It affects 5% of individuals with severe malaria and often culminates in severe clinical complications including severe malarial anemia (SMA), hemoglobinuria, and cerebral malaria (CM) [2]. Malaria is considered a global health threat, with rapidly increasing numbers affecting over 100 nations and more than 219 million people globally. It is estimated that almost 50% of the global population is susceptible to malaria and nearly 1 million individuals die annually from the infection [3].

Saudi Arabia is one of the largest countries in the Middle East with a population of over 32 million. The implementation of malaria control measures in Saudi Arabia over the past 50 years, in collaboration with the World Health Organization (WHO), has effectively reduced the magnitude of risk throughout the nation [4–6]. In 2016, Saudi Arabia was included in the “*Eliminating Countries for 2020* (*E*-*2020*)” initiative of the WHO to achieve the goal of a malaria-free country by 2020 [3]. According to the WHO country profile (2017), there are currently 64 active foci of malaria in Saudi Arabia. The number of individuals living in the active foci is 173,000 (1%) with the majority (32.8 million (99%)), living in malaria-free areas. The predominant *Plasmodium* species causing the majority of the malaria cases is *P*. *falciparum* (97%) followed by *P*. *vivax* (2%).

It is known that the host genetic polymorphisms play an important role in the variation of malaria severity associated with *P*. *falciparum* infection. Also known is the fact that malaria parasites have a robust selective pressure on human genetics, particularly in regions where malaria is endemic [7, 8]. Various disease phenotypes caused by *P*. *falciparum*are influenced by host genetic factors and assist in establishing the pathological mechanisms underlying the vulnerability to the infection [9]. For example, single-nucleotide polymorphisms (SNPs) in the tumor necrosis factor-alpha (TNF*α*) gene are associated with an increase in SMA in *P*. *falciparum*-infected patients [10]. SNPs in the TNF*α* gene have been shown to increase the susceptibility to severe malaria [11].

Interleukin-22 (IL-22) has been classified as a cytokine of the IL-20 subfamily. The *IL*-*22* gene is positioned at chromosome 12q15. Both innate and adaptive arms of the immune system produce IL-22, including *αβ* T, *γδ* T, NKT, and innate lymphoid cells (ILCs). Certain nonhematopoietic and myeloid cells can also release IL-22 [12]. IL-22 affects the outcome of several diseases, such as multiple sclerosis [13], psoriasis [14], inflammatory bowel disease (IBD) [14], Guillain-Barré syndrome (GBS) [15], and West Nile encephalitis [16].

Polymorphisms in the *IL*-*22* gene are associated with several diseases such as ulcerative colitis (UC) [17], psoriasis vulgaris [18], bladder cancer [19], and systemic lupus erythematosus (SLE) [20]. It has been suggested that the variations contribute to malaria pathogenesis. Koch et al. found an association between a IL-22 polymorphism and vulnerability to CM, with other variations associated with protection [21] and an IL-22 polymorphism, namely rs2227473, was associated with childhood CM pathogenesis [22]. In experimental *P*. *chabaudi* malaria infection in mice, a higher production of IL-22 was observed in the liver, which is important as it suggests that this cytokine is an essential factor in the protection against the lethal infection caused by *P*. *chabaudi* [23]. Such a finding could suggest an important role for IL-22 in *P*. *falciparum*-associated malaria and pathogenesis. Therefore, this report investigates the plausible role of variations at the *IL*-*22* gene in the complications associated with *P*. *falciparum* infection in a Saudi Arabian population.

## 2. Materials and Methods

### 2.1. Study Design

Venous blood was collected from 250 *P*. *falciparum*-infected patients admitted to the Malaria Center in the Jazan region, located in the southwest of Saudi Arabia. The study also included 200 randomly selected uninfected healthy individuals as a control group. A thick blood film from each infected patient was used to determine the parasite density according to the “plus system” scale, described in the WHO manual [24]. Based on the parasite density, patients were categorized in four groups: Group I: 1-10 parasites per 100 thick film fields, Group II: 11-100 parasites per 100 thick film fields, Group III: 1-10 parasites per single thick film field, and Group IV: more than 10 parasites per single thick film field.

### 2.2. Ethics Statement

This study was approved by the Research Ethics Committee at King Fahad Hospital (KFCH), Jazan (Registry number 041) and conducted in accordance with the Helsinki Declaration of 1975. Informed consent were obtained from all participants prior to their participation in the study; consent for children were given by their legal guardians. To ensure confidentiality, patient data and biological samples were anonymized prior to processing by laboratory staff. Basic demographic and clinical data required to accomplish the objectives of this study were collected and securely retained for all study participants.

### 2.3. SNP Selection and Genotyping

A total of ten SNPs within the *IL*-*22* gene region were selected for genotyping. The IL-22 SNPs included were rs976748, rs1179246, rs2046068, rs1182844, rs2227508, rs2227513, rs2227478, rs2227481, rs2227491, and rs2227483. Candidate SNPs were selected based on the following criteria: (i) a minor allele frequency (MAF) ≥ 5%, as reported by the 1000 genome for the combined populations and (ii) a linkage disequilibrium (LD) threshold (*r*^2^) ≤ 0.8 or (iii) existence of evidence for an association with malaria. Marker information of the SNPs selected is shown in Supplementary [Supplementary-material supplementary-material-1]. Genotyping the SNPs was performed using polymerase chain reaction (PCR)-based direct DNA sequencing.

DNA was extracted using the DNeasy Blood and Tissue kit (Qiagen, Hilden, Germany) according to the manufacturer's protocol and stored at -20°C for further experimentation. The PCR was performed using the GoTaq Green Master Mix PCR (Promega, Madison, WI, USA). Primers flanking SNPs of interest were designed using Primer3 Input (version 0.4.0) (http://bioinfo.ut.ee/primer3-0.4.0/). All primers were tailed with an M13 sequence to facilitate the sequencing of the PCR products. Sequences of the primers and PCR conditions used for each SNP are shown in Supplementary [Supplementary-material supplementary-material-1]. The PCR products were detected by 2% gel electrophoresis to ensure the quality of the PCR products. The amplification products were subjected to direct sequencing using a BigDye Terminator v3.1 Cycle Sequencing Kit (Applied Biosystems, Foster City, CA, USA) according to the manufacturer's instructions. Each PCR product was sequenced in the forward and reverse directions. The sequences were analyzed and edited with the SeqMan Pro 15 Lasergene (DNASTAR, Inc., Madison, WI, USA).

### 2.4. Luciferase Reporter Assays to Monitor the Activity of Gene Expression on IL-22 Promotor

Gene fragments of the promoter region containing IL-22 rs2227513 A/G variants were amplified and cloned in the pGL3 Basic Vector (Promega, Madison, WI, USA). Primers were tailed at the 5′ end with specifying endonuclease recognition sites for the restriction enzymes SmaI and BglII (New England Biolabs, Ipswich, MA, USA). In addition, a few overhang sequences (CGCCTA) were added to facilitate restriction enzyme binding. Primers used for the amplification of the promoter region, PCR conditions, and chromosomal locations are shown in Supplementary [Supplementary-material supplementary-material-1]. PCR amplification for cloning was conducted using the HotStar DNA Polymerase (Qiagen, Hilden, Germany), following the manufacturer's protocol. *Escherichia coli* DH5*α* competent cells were used for colony selection on ampicillin plates. Clones were selected and DNA was extracted using a QIAamp DNA mini kit (Qiagen, Valencia, CA) following the manufacturer's instructions. Extracted plasmids with cloned fragments were transfected into HEK293 cells (Human Embryonic Kidney 293 cells), which were harvested and tested for luciferase activity after 48 hours.

### 2.5. Statistical Analysis

The SNPs were analyzed and measures of LD, haplotype frequencies, and MAFs were calculated using the Haploview software (version 4.0) (Broad Institute of MIT and Harvard, Cambridge, MA, USA). The de Finetti program (http://ihg.gsf.de/cgi-bin/hw/hwa1.pl) was used to conduct a genotypic association test for all the selected variants as the primary test of association. We also performed the allelic, dominant, and recessive model for each variant to explore the possibility of other models of inheritance. The results were expressed as odds ratios (OR) with 95% confidence intervals (95% CI). Testing of deviation from the Hardy-Weinberg equilibrium (HWE) was performed; rs2227513 was excluded from further analyses due to the significant deviation from HWE (*P* value < 0.05). The test of association was considered significant if the *P* value, obtained in a two-tailed test, was <0.05.

## 3. Results

### 3.1. The Association of the IL-22 Genotype and Allele Distribution and Risk of P. falciparum Malaria

The genotype distribution of the SNPs in the IL-22 gene between the *P*. *falciparum*-infected group and control group was investigated. The frequency of the TT genotype of rs2227481 was significantly higher in the control group compared to the malaria group (8.5% versus 2.4%, OR = 0.254; 95% CI 0.097-0.663, *χ*^2^ = 8.9, and *P* = 0.002) (Table 1). In addition, the T allele at variant rs2227481 was statistically higher in the control compared to the malaria group (allele frequency 22.7% versus 16.2%, OR = 0.656; 95% CI 0.470-0.916, *χ*^2^ = 6.17, and *P* = 0.013). We also found that the T allele was associated with a lower risk of *P*. *falciparum* malaria in a recessive model (OR = 0.265; 95% CI 0.102–0.685, *χ*^2^ = 8.52, and *P* = 0.003). For IL-22 variant rs2227483, we identified that the heterozygous AT genotype frequency was significantly higher in the control group in comparison to the patient group (genotype frequency 13% versus 5.2%, OR = 0.375; 95% CI 0.187-0.754, *χ*^2^ = 8.05, and *P* = 0.004).

### 3.2. The Association of the IL-22 Genotype and Allele Distribution and Risk of Advanced P. falciparum Malaria Based on Parasite Density

The malaria group was classified into four cohorts depicting low to high parasite density: I, II, III, and IV. The rs2227481 CT genotype was significantly higher in cohort IV than in cohorts I+II+III and associated with a higher risk of the more severe form of the disease (genotype frequency 33.1% versus 17.8%, OR = 2.208; 95% CI 1.170-4.170, *χ*^2^ = 6.13, and *P* = 0.013) (Table 2). In addition, the T allele was associated with a higher vulnerability to develop advanced *P*. *falciparum* malaria infection in a dominant model (OR = 1.833; 95% CI 1.012-3.322, *χ*^2^ = 4.05, and *P* = 0.044). The IL-22 SNPs were examined in cohort I compared to cohort II+III+IV and cohort I+II compared to cohorts III+IV; however, none of the SNPs showed any statistically significant distribution between the groups (Supplementary Tables [Supplementary-material supplementary-material-1] and [Supplementary-material supplementary-material-1]).

### 3.3. The Association of the IL-22 Genotype and Allele Distribution Based on the Age of Infected Patients

A comparison of the malaria group in the 1-5 year age group with the 6 years and older age group indicated that the frequency of the rs2046068 A allele was statistically lower in the 6 years and older group (67% versus 92.9%, OR = 6.380; 95% CI 0.827-49.203, *χ*^2^ = 4.14, and *P* = 0.044). Also, the rs2046068 A allele was significant in a dominant model (OR = 7.5; 95% CI 0.889-63.242, *χ*^2^ = 4.67, and *P* = 0.030) (Table 3).

Comparing the malaria group in a 1-9 years age group with a 10 years and older age group, the frequency of the rs976748 CT genotype was significantly higher in the 1-9 years age group compared to the other group (genotype frequency 16.7% versus 4.3%, OR = 0.226; 95% CI 0.056-0.910, *χ*^2^ = 5.14, and *P* = 0.023) (Table 4). Under a dominant mode of inheritance, rs976748 was associated with a higher risk of *P*. *falciparum* malaria infection in the 10 years and older age group (OR = 0.249; 95% CI 0.063-0.989, *χ*^2^ = 4.49, and *P* = 0.034). The rs2227478 CT genotype was substantially elevated in the 10 years and older age group malaria compared to the younger age group (genotype frequency 47.8% versus 22.2%, OR = 3.430; 95% CI 1.056-11.138, *χ*^2^ = 4.66, and *P* = 0.030) (Table 4). Similarly, the A allele of SNP rs2046068 was more frequent in the 1-9 years age group (83.3% versus 66.6%, OR = 2.508; 95% CI 1.022-6.154, *χ*^2^ = 4.29, and *P* = 0.038), with the T allele of rs2227483 associated with the 10 years and older age group (85.3% versus 69.4%, OR = 0.390; 95% CI 0.184-0.830, *χ*^2^ = 6.35, and *P* = 0.011).

No relationship was observed between the IL-22 SNPs and the risk of *P*. *falciparum* malaria comparing the malaria group in the 10-20 years age group with the 21 years and older group (Supplementary [Supplementary-material supplementary-material-1]). Finally, we investigated whether the IL-22 gene SNPs significantly differed in frequency between the *P*. *falciparum* malaria group, 1-9 years age group, and 41 years and older age group (Table 5). We found that the IL-22 rs2046068 A allele was significantly associated with a higher risk of *P*. *falciparum* malaria in the 1-9 years age group. In addition, the A allele of rs2046068 was statistically higher in the 1-9 years age group compared to the 41 years and older age group (allele frequency 83.3% versus 65.7%, OR = 2.606; 95% CI 0.995-6.821, *χ*^2^ = 3.99, and *P* = 0.045). No dominant or recessive statistically significant relationship was observed between the two groups. In contrast, the IL-22 rs2227483 T allele was associated with a high susceptibility to develop *P*. *falciparum* malaria in the 41 years and older age group with a higher frequency of the rs2227483 T allele (allele frequency 86.1% versus 69.4%, OR = 0.367; 95% CI 0.150-0.897, *χ*^2^ = 5.07, and *P* = 0.024). For both SNPs, no dominant or recessive relationship was detected.

### 3.4. The Association of the IL-22 Haplotype and Risk of P. falciparum-Associated Malaria

A haplotype analysis was done between the *P*. *falciparum*-infected group and the control group and two blocks were generated. Block 1 comprised the IL-22 polymorphisms rs1179246, rs1182844, and rs976748, with block 2 consisting of IL-22 polymorphisms rs2046068 and rs2227491 (Table 6). One haplotype in block 1 was statistically significant. The haplotype A-T-T involving the alleles of rs1179246, rs1182844, and rs976748 was statistically more frequent in the control group than the malaria group (frequency 41%, *P* = 0.034). In block 2, one haplotype A-G of rs2046068 and rs2227491 was statistically significant and prevalent in the control group but not in the malaria group (Frequency 49.4%, *P* = 0.041).

### 3.5. The Association of the IL-22 Haplotype and Risk of Advanced P. falciparum Malaria Based on Parasite Density

A haplotype analysis was performed between cohort I+II and cohort III+IV of varied parasite density (Table 7). The distribution of only one haplotype was found to be significantly different in the groups. The haplotype C-A-C-A-C-C including alleles of rs1179246, rs1182844, rs2046068, rs2227491, rs2227481, and rs2227478 occurred more frequently in cohort I+II (frequency 13.7%, *P* = 0.036).

### 3.6. The Association of the IL-22 Haplotype and Risk of Malaria Based on Age Groups

A haplotype distribution analysis was done in the malaria group for two age groups, 1-5 years and 6 years and older. The frequency of two haplotypes was found to be either statistically significant or close to significance (Table 8). The haplotype A-G of rs2046068 and rs2227491 was more frequent in the 1-5 years age group (frequency 46.3%, *P* = 0.056). However, the haplotype C-A of rs2046068 and rs2227491 was significantly high in the 6 years and older age group (frequency 31.9%, *P* = 0.043).

A haplotype analysis was also performed between patients in the 1-9 years and 10 years and older age groups. One single haplotype was statistically significant (Table 9). The haplotype C-A of rs2046068 and rs2227491 was elevated in the 10 years and older age group compared with the 1-9 years age group (frequency 31.9%, *P* = 0.041).

Finally, we assessed the haplotype distribution between patients in the 1-9 years and 41 years and older age groups. The distribution of two haplotypes was significantly different in these two groups (Table 10). The haplotype C-A of rs2046068 and rs2227491 was higher in 41 years and older age group (frequency 29.9%, *P* = 0.045).

### 3.7. Functional Analysis of the SNPs Located in the IL-22 Promoter

**Figure 1:**
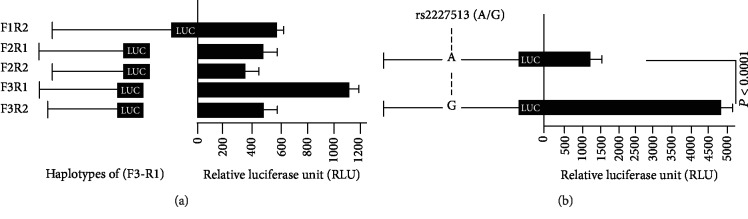
Luciferase activity assay for fragments containing the promoter of *IL*-*22* gene. (a) Nested deletions of the promoter region were generated and cloned. The clones were then transfected in HEK293 cell line for 48 h. Cells were harvested and tested for luciferase activity. (b) Two constructs of fragment F3R1 containing SNP rs2227513 were generated. One clone harbored A allele and the other clone contained G allele of SNP rs2227513. Luciferase activity was performed as shown in (a).

For the delineation of the molecular mechanisms driving the IL-22 gene, nested deletions from the IL-22 promoter were generated (Figure 1(a)) and cloned in a luciferase expression system. The clones were transfected in the HEK293 cell line for 48 hours. Cells were harvested and tested for mean luciferase activity. As demonstrated in Figure 1(a), construct F3R1 containing IL-22 variant rs2227513 showed maximum luciferase activity.

To test whether rs2227513 contributes to the expression of the IL-22, one nucleotide was changed in clone F3/R1 (Figure 1(b)). The IL-22 promoter activity of the variant rs2227513 alleles A and G was measured. The findings indicated that the HEK293 cells transfected with variant rs2227513 G allele had statistically significant higher luciferase activity compared to the A allele (*P* < 0.0001).

## 4. Discussion

Globally, malaria remains one of the major causes of mortality in children younger than 5 years [3]. Recently a malaria vaccine, known as RTS, S/AS01 (RTS, S), was introduced for a pilot study in three African countries, Ghana, Kenya, and Malawi. However, the vaccine is reportedly effective only against *P*. *falciparum* and has an efficacy of 39% [25]. The diverse manifestations of the disease in malaria patients drives the urgency in the search for a vaccine. It is known that there is a significant variation in the clinical symptoms associated with malaria. While some patients would die of severe malaria infection, others would have an uncomplicated infection [26]. Such disease variation suggests that in addition to the parasite and environmental influence, the genetic characteristics of the host is central in determining the clinical outcome in *P*. *falciparum*-infected patients. The understanding of genetic differences in populations substantially transformed the development of vaccines in general and assisted in developing the most recent malaria vaccine [25, 27].

In this study, we examined the frequency of nine SNPs (rs976748, rs1179246, rs2046068, rs1182844, rs2227508, rs2227478, rs2227481, rs2227491, and rs2227483) in the *IL*-*22* gene in *P*. *falciparum* malaria infection. The aim was to determine whether there is an association in terms of vulnerability or protection as indicated by the level of parasite density or the age group distribution. IL-22 is suggested to have a protective role in severe anemia-associated malaria infection, as it has been reported that IL-22 is elevated in children infected with *P*. *falciparum* [28]. Only a few studies have investigated the role of IL-22 SNPs in malaria [21, 22, 29]. The first report of the involvement of IL-22 SNPs in malaria found two IL-22 SNPs in West African children infected with *P*. *falciparum* malaria; one correlated with protection against SM (rs2227491) and the second with susceptibility to CM (rs2227478) [21]. However, both SNPs were not significant in our population for any of the tested traits. Another study in Nigerian and Malawian populations found that the T allele of two IL-22 variants, rs2227476 and rs2227473, in the promoter region is associated with CM [22]. Notably, rs2227476 is in high LD (*r*^2^ = 0.92) with rs2227481 and is significantly associated with the risk of *P*. *falciparum* malaria in our population. There is a dearth of data to study the role of the *IL*-*22* gene with vulnerability or protection, parasite density and age-related risk of *P*. *falciparum* malaria infection.

In the present study, we strengthen the possible relationship between the *IL*-*22* rs2227481 T allele with protection against *P*. *falciparum* malaria infection in a recessive mode of inheritance, with a similar association found for individuals with a homozygous TT genotype. It was also found that the C allele of this SNP is associated with an increased risk of malaria infection. Our highest significant SNP (rs2227481) is in high LD with rs2227476, previously linked to a predisposition to childhood CM in an African population, potentially by altering the binding site for several transcription factors as suggested by the *in silico* analysis [22]. Our study supports the evidence of the role of *IL*-*22* in the clinical manifestation of *P*. *falciparum*-associated malaria. It has been reported that the IL-22 rs2227481 polymorphism plays a crucial role in effector T cell pathways [30]. Exploring the influence of IL-22 rs2227481 on parasite density and age-related risk of malaria infection, we found that the IL-22 rs2227481 homozygous CC genotype, in a dominant mode of inheritance, is associated with a higher *P*. *falciparum* parasite density in the Saudi Arabian population. The rs2227481 T allele was associated with a low parasite density in a dominant model. However, no relationship was observed for an age-related risk of the infection. The results indicate that the rs2227481 T and homozygous TT genotype may be important in protecting against *P*. *falciparum* malaria infection.

The IL-22 variant rs2227483 is located within putative binding sites for the aryl-hydrocarbon receptor (AhR-ARNT) complex, and it has been suggested that this complex transcription factor induce IL-22 expression [31]. The AhR-ARNT receptor complex interacts with miscellaneous ligands and is a key in regulating IL-22 production [22]. For the IL-22 rs2227483 polymorphism, the heterozygous AT genotype was significantly associated with protection against *P*. *falciparum* infection. Zhang et al. demonstrated that the A and T alleles of IL-22 rs2227483 were significantly elevated in controls compared to tuberculosis (TB) cases and suggested that rs2227483 A and T alleles are associated with increased production of IL-22 decreasing vulnerability to TB infection [32]. Notably, others have found that the rs2227483 genotype TT is statistically associated with early-onset psoriasis through the high production of IL-22 cytokine [31]. Although we did not find any association between the rs2227483 TT genotype and *P*. *falciparum* infection, the malaria group possessed a higher TT genotype compared to the controls. Our findings and literature suggest that the variant rs2227483 TT genotype could have a role in the altered IL-22 expression and increased risk of malaria infection.

As parasite density is associated with a more severe outcome in *P*. *falciparum* malaria [33, 34], we analyzed our data in relation to parasite density. The only variant that had a statistically significant distribution is rs2227481 when cohorts I+II+III were compared to cohort IV. This SNP is located upstream of the *IL*-*22* gene and could influence the expression levels of the protein; however, the conclusion should be substantiated by experimental evidence.

The *IL*-*22* gene polymorphism rs2046068 was not statistically associated with either protection or risk of malaria infection or the level of parasite density. Similarly, Weger et al. did not find any relationship of the rs2046068 genotype and haplotype analysis with chronic plaque psoriasis [35]. In the current study, the rs2046068 AA genotype was higher in the control group. Song et al. also showed an elevated rs2046068 AA genotype in the control group in contrast to a group with autoimmune thyroid disease (AITD), Graves' disease (GD), and Hashimoto's thyroiditis (HT). However, the relationship was not statistically significant [36]. We found that the rs2046068 A allele in the IL-22 gene was statistically high under a dominant mode of inheritance in the malaria group within the 1-5 years age group compared to the 6 years and older age group. Conversely, the frequency of the rs2046068 C allele was predominant in the malaria group, 6 years and older, in a dominant inheritance model.

The haplotype distribution analysis at the *IL*-*22* gene demonstrated statistically significant frequencies between the control and *P*. *falciparum*-infected malaria groups. We detected a high frequency of the rs1179246-rs1182844-rs976748 A-T-T haplotype in the control group compared to the malaria group, suggesting the role of the A-T-T haplotype in protecting against *P*. *falciparum* infection. Similarly, an elevated frequency of the rs2046068-rs2227491 A-G haplotype was observed in the control group. Wang et al. did not find any association between the rs2227491 and serum IL-22 levels of the control group in SLE cases in a Chinese population [20]. With the haplotype analysis for the level of parasite density when comparing cohort I+II with III+IV, the results showed a significantly higher frequency of the rs1179246-rs1182844-rs2046068-rs2227491-rs2227481-rs2227478 C-A-C-A-C-C haplotype in the I+II cohort. The contribution of these haplotypes to *P*. *falciparum*-associated clinical outcomes and the function of IL-22 need to be experimentally substantiated.

The variant rs2227513 is positioned at the first intron of the *IL*-*22* gene and it is also part of the promoter region of the *IL*-*22* gene. Studies suggest that introns are imperative modulators of gene functions such as RNA editing [37, 38]. In the current study, the IL-22 rs2227513 G allele was associated with higher expression levels of the IL-22 cytokine, as evidenced by statistically higher luciferase reporter gene activity. Although this SNP was excluded from the test of association analysis due to the significant deviation from HWE, it is almost a complete LD (*r*^2^ = 0.98) with rs976748, associated with a higher risk of *P*. *falciparum* malaria infection.

Several studies have shown that variations at the IL-22 gene could contribute to the outcome of different diseases. Mastelic et al. demonstrated a shielding role of IL-22 cytokine against *P*. *chabaudi*-induced liver damage [23]. Also, Hu et al. reported a high possibility of HIV infection with the rs2227513 AG genotype [39]. It was also suggested that there is a positive association between the rs2227513 AG genotype and susceptibility to SLE [20]. The latter study showed that the levels of the IL-22 cytokine were statistically less in the AG genotype in contrast to AA genotype, signifying that the rs2227513 AG genotype increases the risk of SLE by reducing the IL-22 cytokine levels [20]. Our findings indicate a potential pathological role of the rs2227513 G allele in malaria infection, evidenced by the higher promoter activity in the luciferase assay.

This study suggests that variants in the *IL*-*22* gene may produce different roles in the vulnerability to infection in populations with diverse ethnic backgrounds. A plausible explanation of this disparity could be environmental or other genetic elements. As we did not investigate all the *IL*-*22* gene variants, future case control studies are necessary to confirm the findings of this study and to explore additional links between IL-22 SNPs and the clinical outcome of *P*. *falciparum*-associated malaria. It is essential to reproduce a similar study in other populations with a different genetic background to replicate our findings in a larger sample size. In addition, studies to outline the molecular or cellular mechanisms underlying the role of IL-22 in malaria are recommended.

## 5. Conclusion

Our data shows that the IL-22 rs2227481 TT genotype and T allele and the rs2227483 AT genotype and A allele may be associated with protection against *P*. *falciparum* malaria. The IL-22 promoter polymorphism in rs2227513 G allele appears to be associated with higher expression levels of IL-22, which could play a role in the protection against the disease. These findings will increase the current understanding of the mechanisms involved in the pathogenesis of malaria infection.

## Figures and Tables

**Table 1:** Genotype distribution and allele frequency of IL-22 SNPs among malaria patients compared to healthy control subjects.

SNPs	Genotype/allele distribution	Control	Patients	OR (95% CI)	*χ* ^2^	*P* value
rs976748	TT	193 (96.5%)	236 (94.4%)	Ref		
	CT	6 (3%)	13 (5.2%)	1.772 (0.661-4.749)	1.33	0.249
	CC	1 (0.5%)	1 (0.4%)	0.818 (0.051-13.160)	0.02	0.886
	T	392 (98%)	485 (97%)	1.515 (0.636-3.611)	0.89	0.344
	C	8 (2%)	15 (3%)			
	CC+CT vs. TT			1.636 (0.647-4.133)	1.1	0.293
	CC vs. CT+TT			1.251 (0.078-20.130)	0.03	0.874

rs1179246	CC	55 (27.5%)	86 (34.4%)	Ref		
	AC	98 (49%)	112 (44.8%)	0.731 (0.474-1.128)	2.01	0.155
	AA	47 (23.5%)	52 (20.8%)	0.708 (0.421-1.190)	1.71	0.191
	C	208 (52%)	284 (56.8%)	0.824 (0.633-1.073)	2.07	0.150
	A	192 (48%)	216 (43.2%)			
	AA+AC vs. CC			0.723 (0.482-1.085)	2.46	0.116
	AA vs. AC+CC			0.855 (0.547-1.338)	0.47	0.492

rs2046068	AA	104 (52%)	114 (45.6%)	Ref		
	AC	76 (38%)	111 (44.4%)	1.332 (0.898-1.977)	2.03	0.153
	CC	20 (10%)	25 (10%)	1.140 (0.598-2.174)	0.16	0.689
	A	284 (71%)	339 (67.8%)	1.163 (0.873-1.548)	1.07	0.301
	C	116 (29%)	161 (32.2%)			
	CC+AC vs. AA			1.292 (0.890-1.876)	1.82	0.177
	CC vs. AC+AA			1.000 (0.538-1.859)	0	1

rs1182844	TT	83 (41.5%)	90 (36%)	Ref		
	AT	95 (47.5%)	120 (48%)	1.165 (0.779-1.741)	0.55	0.456
	AA	22 (11%)	40 (16%)	1.677 (0.921-3.054)	2.88	0.089
	T	261 (65.3%)	300 (60%)	1.252 (0.953-1.644)	2.61	0.106
	A	139 (34.5%)	200 (40%)			
	AA+AT vs. TT			1.261 (0.861-1.848)	1.42	0.233
	AA vs. AT+TT			1.541 (0.883-2.690)	2.34	0.126

rs2227508	AA	140 (70)	166 (66.4%)	Ref		
	AT	56 (28)	71 (28.4%)	1.069 (0.705-1.621)	0.1	0.752
	TT	4 (2)	13 (5.2%)	2.741 (0.874-8.596)	3.22	0.072
	A	336 (84)	403 (80.6%)	1.264 (0.893-1.788)	1.75	0.186
	T	64 (16)	97 (19.4%)			
	TT+AT vs. AA			1.181 (0.791-1.762)	0.66	0.415
	TT vs. AT+AA			2.333 (0.750-7.264)	3.13	0.076

rs2227478	TT	84 (42%)	100 (40%)	Ref		
	CT	82 (41%)	115 (46%)	1.178 (0.785-1.767)	0.63	0.428
	CC	34 (17%)	35 (14%)	0.865 (0.497-1.505)	0.26	0.606
	T	250 (62.5%)	315 (63%)	0.979 (0.746-1.285)	0.02	0.877
	C	150 (37.5%)	185 (37%)			
	CC+CT vs. TT			1.086 (0.744-1.585)	0.18	0.668
	CC vs. CT+TT			0.795 (0.476-1.328)	0.77	0.380

rs2227481	CC	126 (63%)	175 (70%)	Ref		
	CT	57 (28.5%)	69 (27.6%)	0.872 (0.573-1.325)	0.41	0.520
	TT	17 (8.5%)	6 (2.4%)	0.254(0.097-0.663)	8.9	0.002
	C	309 (77.3%)	419 (83.8%)	0.656 (0.470-0.916)	6.17	0.013
	T	91 (22.7%)	81 (16.2%)			
	TT+CT vs. CC			0.730 (0.492-1.083)	2.46	0.116
	TT vs. CT+CC			0.265 (0.102-0.685)	8.52	0.003

rs2227491	AA	60 (30%)	72 (28.8%)	Ref		
	AG	83 (41.5%)	123 (49.2%)	1.235 (0.794-1.920)	0.88	0.348
	GG	57 (28.5%)	55 (22%)	0.804 (0.485-1.332)	0.72	0.396
	A	203 (50.8%)	267 (53.4%)	0.899 (0.691-1.170)	0.63	0.429
	G	197 (49.2%)	233 (46.6%)			
	GG+AG vs. AA			1.060 (0.705-1.593)	0.08	0.781
	GG vs. AG+AA			0.708 (0.461-1.086)	2.51	0.113

rs2227483	TT	153 (76.5%)	204 (81.6%)	Ref		
	AT	26 (13%)	13 (5.2%)	0.375 (0.187-0.754)	8.05	0.004
	AA	21 (10.5%)	33 (13.2%)	1.179 (0.656-2.117)	0.3	0.582
	T	332 (83%)	421 (84.2%)	0.916 (0.643-1.306)	0.23	0.628
	A	68 (17%)	79 (15.8%)			
	AA+AT vs. TT			0.734 (0.465-1.160)	1.76	0.184
	AA vs. AT+TT			1.296 (0.724-2.319)	0.77	0.381

**Table 2:** Genotype distribution and allele frequency of IL-22 SNPs in group I+II+III of parasite density compared to group IV.

SNPs	Genotype/allele distribution	Group I+II+III	Group IV	OR (95% CI)	*χ* ^2^	*P* value
rs976748	TT	86 (95.6%)	150 (93.8%)	Ref		
	CT	4 (4.4%)	9 (5.6%)	1.290 (0.386-4.314)	0.17	0.678
	CC	0 (0%)	1 (0.6%)	1.724 (0.069-42.790)	0.57	0.449
	T	176 (97.8%)	309 (96.6%)	1.566 (0.491-4.993)	0.58	0.444
	C	4 (2.2%)	11 (3.4%)			
	CC+CT vs. TT			1.433 (0.436-4.709)	0.36	0.551
	CC vs. CT+TT			0.587 (0.024-14.571)	0.56	0.452

rs1179246	CC	28 (31.1%)	57 (35.6%)	Ref		
	AC	43 (47.8%)	69 (43.1%)	0.788 (0.436-1.424)	0.62	0.429
	AA	19 (21.1%)	34 (21.3%)	0.879 (0.427-1.808)	0.12	0.725
	C	99 (55%)	183 (57.2%)	0.915 (0.633-1.322)	0.22	0.635
	A	81 (45%)	137 (42.8%)			
	AA+AC vs. CC			0.816 (0.470-1.416)	0.52	0.469
	AA vs. AC+CC			1.008 (0.536-1.898)	0	0.979

rs2046068	AA	39 (43.3%)	75 (46.9%)	Ref		
	AC	44 (48.9%)	67 (41.9%)	0.792 (0.460-1.362)	0.71	0.398
	CC	7 (7.8%)	18 (11.2%)	1.337 (0.515-3.475)	0.36	0.550
	A	122 (67.8%)	217 (67.8%)	0.998 (0.675-1.476)	0	0.993
	C	58 (32.2%)	103 (32.2%)			
	CC+AC vs. AA			0.867 (0.515-1.458)	0.29	0.589
	CC vs. AC+AA			1.503 (0.603-3.749)	0.77	0.379

rs1182844	TT	27 (30%)	63 (39.4%)	Ref		
	AT	46 (51.1%)	74 (46.2%)	0.689(0.385-1.234)	1.57	0.209
	AA	17 (18.9%)	23 (14.4%)	0.580 (0.268-1.255)	1.93	0.164
	T	100 (55.6%)	200 (62.5%)	0.750 (0.518-1.087)	2.31	0.128
	A	80 (44.4%)	120 (37.5%)			
	AA+AT vs. TT			0.660 (0.380-1.145)	2.2	0.138
	AA vs. AT+TT			0.721 (0.362-1.435)	0.87	0.350

rs2227508	AA	57 (63.3%)	109 (68.1%)	Ref		
	AT	26 (28.9%)	45 (28.1%)	0.905 (0.507-1.616)	0.11	0.735
	TT	7 (7.8%)	6 (3.8%)	0.448 (0.144-1.397)	2	0.157
	A	140 (77.8%)	263 (82.2%)	0.759 (0.482-1.194)	1.43	0.231
	T	40 (22.2%)	57 (17.8%)			
	TT+AT vs. AA			0.808 (0.470-1.390)	0.59	0.441
	TT vs. AT+AA			0.462 (0.150-1.420)	1.9	0.168

rs2227478	TT	35 (38.9%)	65 (40.6%)	Ref		
	CT	38 (42.2%)	77 (48.1%)	1.091 (0.620-1.921)	0.09	0.762
	CC	17 (18.9%)	18 (11.3%)	0.570(0.261-1.244)	2.02	0.155
	T	108 (60%)	207 (64.7%)	0.819 (0.562-1.193)	1.09	0.297
	C	72 (40%)	113 (35.3%)			
	CC+CT vs. TT			0.930 (0.548-1.577)	0.07	0.787
	CC vs. CT+TT			0.544 (0.265-1.119)	2.79	0.094

rs2227481	CC	70 (77.8%)	105 (65.6%)	Ref		
	CT	16 (17.8%)	53 (33.1%)	2.208 (1.170-4.170)	6.13	0.013
	TT	4 (4.4%)	2 (1.3%)	0.333 (0.059-1.869)	1.71	0.191
	C	156 (86.7%)	263 (82.2%)	1.409 (0.841-2.361)	1.7	0.191
	T	24 (13.3%)	57 (17.8%)			
	TT+CT vs. CC			1.833 (1.012-3.322)	4.05	0.044
	TT vs. CT+CC			0.272 (0.0488-1.516)	2.51	0.113

rs2227491	AA	24 (26.7%)	48 (30%)	Ref		
	AG	49 (54.4%)	74 (46.2%)	0.755 (0.411-1.388)	0.82	0.365
	GG	17 (18.9%)	38 (23.8%)	1.118 (0526-2.373)	0.08	0.772
	A	97 (53.9%)	170 (53.1%)	1.031 (0.715-1.487)	0.03	0.869
	G	83 (46.1%)	150 (46.9%)			
	GG+AG vs. AA			0.848 (0.477-1.510)	0.31	0.576
	GG vs. AG+AA			1.338 (0.704-2.540)	0.79	0.373

rs2227483	TT	73 (81.1%)	131 (81.9%)	Ref		
	AT	4 (4.4%)	9 (5.6%)	1.254 (0.373-4.213)	0.13	0.714
	AA	13 (14.5%)	20 (12.5%)	0.857 (0.403-1.823)	0.16	0.689
	T	150 (83.3%)	271 (84.7%)	0.904 (0.550-1.485)	0.16	0.690
	A	30 (16.7%)	49 (15.3%)			
	AA+AT vs. TT			0.951 (0.490-1.846)	0.02	0.881
	AA vs. AT+TT			0.846 (0.399-1.794)	0.19	0.662

**Table 3:** Genotype distribution and allele frequency of IL-22 SNPs in group 1-5 years of age compared to 6 years and above age group.

SNPs	Genotype/allele distribution	1-5 years	6 years and above	OR (95% CI)	*χ* ^2^	*P* value
rs976748	TT	7 (100%)	229 (94.3%)	Ref		
	CT	0 (0%)	13 (5.3%)	0.882 (0.048-16.276)	0.4	0.528
	CC	0 (0%)	1 (0.4%)	0.098 (0.004-2.611)	0.03	0.861
	T	14 (100%)	471 (97%)	0.953 (0.054-16.717)	0.45	1
	C	0 (0%)	15 (3%)			
	CC+CT vs. TT			0.948 (0.052-17.423)	0.43	0.513
	CC vs. CT+TT			0.093 (0.004-2.471)	0.03	0.864

rs1179246	CC	2 (28.6%)	85 (35%)	Ref		
	AC	5 (71.4%)	107 (44%)	0.504 (0.095-2.660)	0.68	0.410
	AA	0 (0%)	51 (21%)	3.012 (0.142-63.975)	1.19	0.275
	C	9 (64.3%)	277 (57%)	1.358 (0.449-4.112)	0.3	0.586
	A	5 (35.7%)	209 (43%)			
	AA+AC vs. CC			0.744 (0.141-3.914)	0.12	0.725
	AA vs. AC+CC			4.013 (0.225-71.433)	1.85	0.174

rs2046068	AA	6 (85.7%)	108 (44.4%)	Ref		
	AC	1 (14.3%)	110 (45.3%)	6.111 (0.724-51.609)	3.55	0.059
	CC	0 (0%)	25 (10.3%)	3.055 (0.167-56.007)	1.38	0.240
	A	13 (92.9%)	326 (67%)	6.380 (0.827-49.203)	4.14	0.044
	C	1 (7.1%)	160 (33%)			
	CC+AC vs. AA			7.500(0.889-63.242)	4.67	0.030
	CC vs. AC+AA			1.751 (0.097-31.562)	0.8	0.371

rs1182844	TT	3 (42.9%)	87 (35.8%)	Ref		
	AT	4 (57.1%)	116 (47.7%)	1.00 (0.218-4.584)	0	1
	AA	0 (0%)	40 (16.5%)	3.240 (0.164-64.205)	1.36	0.242
	T	10 (71.4%)	290 (59.7%)	1.690 (0.523-5.464)	0.78	0.375
	A	4 (28.6%)	196 (40.3%)			
	AA+AT vs. TT			1.345 (0.294-6.147)	0.15	0.701
	AA vs. AT+TT			2.985 (0.167-53.317)	1.37	0.241

rs2227508	AA	6 (85.7%)	160 (65.8%)	Ref		
	AT	1 (14.3%)	70 (28.8%)	2.625 (0.310-22.212)	0.84	0.358
	TT	0 (0%)	13 (5.4%)	1.093 (0.058-20.469)	0.49	0.485
	A	13 (92.9%)	390 (80.2%)	3.200 (0.414-24.762)	1.38	0.323
	T	1 (7.1%)	96 (19.8%)			
	TT+AT vs. AA			3.112 (0.369-26.284)	1.2	0.272
	TT vs. AT+AA			0.879 (0.047-16.206)	0.4	0.529

rs2227478	TT	5 (71.4%)	95 (39.1%)	Ref		
	CT	2 (28.6%)	113 (46.5%)	2.974 (0.564-15.677)	1.81	0.179
	CC	0 (0%)	35 (14.4%)	4.089 (0.220-75.860)	1.82	0.177
	T	12 (85.7%)	303 (62.3%)	3.624 (0.802-16.374)	3.19	0.074
	C	2 (14.3%)	183 (37.7%)			
	CC+CT vs. TT			3.895 (0.741-20.482)	2.96	0.085
	CC vs. CT+TT			2.554 (0.143-45.516)	1.17	0.278

rs2227481	CC	6 (85.7%)	169 (69.5%)	Ref		
	CT	1 (14.3%)	68 (28%)	2.414 (0.285-20.430)	0.7	0.404
	TT	0 (0%)	6 (2.5%)	0.499 (0.025-9.827)	0.21	0.644
	C	13 (92.9%)	406 (83.5%)	2.562 (0.330-19.859)	0.87	0.710
	T	1 (7.1%)	80 (16.5%)			
	TT+CT vs. CC			2.627 (0.311-22.209)	0.85	0.357
	TT vs. CT+CC			0.411 (0.0211-7.977)	0.18	0.673

rs2227491	AA	3 (42.9%)	72 (29.6%)	Ref		
	AG	4 (57.1%)	119 (49%)	1.240 (0.270-5.698)	0.08	0.782
	GG	0 (0%)	52 (21.4%)	5.069 (0.256-100.237)	2.13	0.144
	A	10 (71.4%)	263 (54.1%)	2.120 (0.656-6.852)	1.65	0.199
	G	4 (28.6%)	223 (45.9%)			
	GG+AG vs. AA			1.781 (0.389-8.161)	0.57	0.451
	GG vs. AG+AA			4.112 (0.231-73.184)	1.89	0.169

rs2227483	TT	5 (71.4%)	199 (81.9%)	Ref		
	AT	0 (0%)	13 (5.3%)	0.744 (0.039-14.180)	0.33	0.567
	AA	2 (28.6%)	31 (12.8%)	0.389 (0.072-2.096)	1.29	0.255
	T	10 (71.4%)	411 (84.6%)	0.456 (0.139-1.493)	1.77	0.252
	A	4 (28.6%)	75 (15.4%)			
	AA+AT vs. TT			0.553 (0.104-2.942)	0.5	0.481
	AA vs. AT+TT			0.365 (0.068-1.966)	1.49	0.222

**Table 4:** Genotype distribution and allele frequency of IL-22 SNPs in group 1-9 years of age compared to 10 years and above age group.

SNPs	Genotype/allele distribution	1-9 years	10 years and above	OR (95% CI)	*χ* ^2^	*P* value
rs976748	TT	15 (83.3%)	221 (95.3%)	Ref		
	CT	3 (16.7%)	10 (4.3%)	0.226 (0.056-0.910)	5.14	0.023
	CC	0 (0%)	1 (0.4%)	0.210 (0.008-5.370)	0.07	0.794
	T	33 (91.7%)	452 (97.4%)	0.292 (0.079-1.086)	3.79	0.085
	C	3 (8.3%)	12 (2.6%)			
	CC+CT vs. TT			0.249 (0.063-0.989)	4.49	0.034
	CC vs. CT+TT			0.240 (0.009-6.094)	0.08	0.780

rs1179246	CC	5 (27.8%)	80 (34.5%)	Ref		
	AC	8 (44.4%)	104 (44.8%)	0.812 (0.256-2.578)	0.12	0.724
	AA	5 (27.8%)	48 (20.7%)	0.600 (0.165-2.180)	0.61	0.433
	C	18 (50%)	264 (56.9%)	0.758 (0.384-1.493)	0.65	0.421
	A	18 (50%)	200 (43.1%)			
	AA+AC vs. CC			0.731 (0.252-2.123)	0.33	0.562
	AA vs. AC+CC			10.678 (0.231-1.996)	0.5	0.478

rs2046068	AA	12 (66.7%)	102 (44%)	Ref		
	AC	6 (33.3%)	105 (45.3%)	2.059 (0.745-5.693)	2	0.156
	CC	0 (0%)	25 (10.7%)	6.220 (0.356-108.579)	2.88	0.089
	A	30 (83.3%)	309 (66.6%)	2.508 (1.022-6.154)	4.29	0.038
	C	6 (16.7%)	155 (33.4%)			
	CC+AC vs. AA			2.549(0.925-7.024)	3.47	0.062
	CC vs. AC+AA			4.547 (0.266-77.748)	2.16	0.142

rs1182844	TT	7 (38.9%)	83 (35.8%)	Ref		
	AT	11 (61.1%)	109 (41%)	0.836 (0.311-2.248)	0.13	0.721
	AA	0 (0%)	40 (17.2%)	7.275 (0.405-130.544)	3.29	0.069
	T	25 (69.4%)	275 (59.3%)	1.562 (0.751-3.251)	1.44	0.229
	A	11 (30.6%)	189 (40.7%)			
	AA+AT vs. TT			1.142 (0.427-3.059)	0.07	0.790
	AA vs. AT+TT			7.784 (0.459-131.835)	3.69	0.054

rs2227508	AA	14 (77.8%)	152 (65.5%)	Ref		
	AT	4 (22.2%)	67 (28.9%)	1.543 (0.490-4.861)	0.56	0.456
	TT	0 (0%)	13 (5.6%)	2.567 (0.145-45.441)	1.19	0.275
	A	32 (88.9%)	371 (80%)	2.005 (0.692-5.811)	1.7	0.191
	T	4 (11.1%)	93 (20%)			
	TT+AT vs. AA			1.842 (0.587-5.781)	1.13	0.288
	TT vs. AT+AA			2.276 (0.130-39.829)	1.06	0.302

rs2227478	TT	11 (61.1%)	89 (38.4%)	Ref		
	CT	4 (22.2%)	111 (47.8%)	3.430 (1.056-11.138)	4.66	0.030
	CC	3 (16.7%)	32 (13.8%)	1.318 (0.346-5.030)	0.16	0.685
	T	26 (72.2%)	289 (62.3%)	1.574 (0.741-3.343)	1.42	0.234
	C	10 (27.8%)	175 (37.7%)			
	CC+CT vs. TT			2.525 (0.944-6.754)	3.6	0.057
	CC vs. CT+TT			0.800 (0.219-2.919)	0.11	0.735

rs2227481	CC	13 (72.2%)	162 (69.8%)	Ref		
	CT	4 (22.2%)	65 (28%)	1.304 (0.410-4.147)	0.2	0.652
	TT	1 (5.6%)	5 (2.2%)	0.401 (0.044-3.695)	0.69	0.404
	C	30 (83.3%)	389 (83.8%)	0.964(0.388-2.397)	0.01	0.937
	T	6 (16.7%)	75 (16.2%)			
	TT+CT vs. CC			1.123 (0.386-3.271)	0.05	0.830
	TT vs. CT+CC			0.374 (0.041-3.389)	0.82	0.363

rs2227491	AA	5 (27.7%)	71 (30.6%)	Ref		
	AG	12 (66.7%)	111 (47.8%)	0.651 (0.220-1.928)	0.61	0.435
	GG	1 (5.6%)	50 (21.6%)	3.52 (0.399-31.065)	1.45	0.229
	A	22 (61.1%)	253 (54.5%)	1.311 (0.654-2.625)	0.59	0.444
	G	14 (38.9%)	211 (45.5%)			
	GG+AG vs. AA			0.872(0.300-2.539)	0.06	0.801
	GG vs. AG+AA			4.670 (0.607-35.952)	2.63	0.104

rs2227483	TT	12 (66.7%)	192 (82.8%)	Ref		
	AT	1 (5.6%)	12 (5.2%)	0.750 (0.090-6.259)	0.07	0.789
	AA	5 (27.7%)	28 (12%)	0.350 (0.115-1.068)	3.67	0.055
	T	25 (69.4%)	396 (85.3%)	0.390 (0.184-0.830)	6.35	0.011
	A	11 (30.6%)	68 (14.7%)			
	AA+AT vs. TT			0.417 (0.148-1.176)	2.88	0.089
	AA vs. AT+TT			0.357 (0.118-1.077)	3.6	0.057

**Table 5:** Genotype distribution and allele frequency of IL-22 SNPs in group 1-9 years of age compared to 41 years and above age group.

SNPs	Genotype/allele distribution	1-9 years	41 years and above	OR (95% CI)	*χ* ^2^	*P* value
rs976748	TT	15 (83.3%)	49 (90.7%)	Ref		
	CT	3 (16.7%)	5 (9.3%)	0.510 (0.109-2.389)	0.75	0.386
	CC	0 (0%)	0 (0%)	0.313 (0.006-16.446)	Nan	1
	T	33 (91.7%)	103 (95.4%)	0.534 (0.121-2.355)	0.71	0.412
	C	3 (8.3%)	5 (4.6%)			
	CC+CT vs. TT			0.510 (0.109-2.389)	0.75	0.386
	CC vs. CT+TT			0.339 (0.007-17.723)	Nan	1

rs1179246	CC	5 (27.8%)	25 (46.3%)	Ref		
	AC	8 (44.4%)	16 (29.6%)	0.400 (0.111-1.441)	2.03	0.154
	AA	5 (27.8%)	13 (24.1%)	0.520 (0.127-2.128)	0.84	0.358
	C	18 (50%)	66 (61.1%)	0.636 (0.298-1.360)	1.37	0.241
	A	18 (50%)	42 (38.9%)			
	AA+AC vs. CC			0.446 (0.140-1.426)	1.9	0.167
	AA vs. AC+CC			0.824 (0.247-2.752)	0.1	0.753

rs2046068	AA	12 (66.7%)	25 (46.3%)	Ref		
	AC	6 (33.3%)	21 (38.9%)	1.680 (0.538-5.247)	0.8	0.369
	CC	0 (0%)	8 (14.8%)	8.333 (0.444-156.275)	3.54	0.059
	A	30 (83.3%)	71 (65.7%)	2.606 (0.995-6.821)	3.99	0.045
	C	6 (16.7%)	37 (34.3%)			
	CC+AC vs. AA			2.320(0.760-7.085)	2.24	0.134
	CC vs. AC+AA			6.763 (0.371-123.24)	3	0.083

rs1182844	TT	7 (38.9%)	20 (37%)	Ref		
	AT	11 (61.1%)	22 (40.7%)	0.700 (0.227-2.155)	0.39	0.533
	AA	0 (0%)	12 (22.2%)	9.146 (0.480-174.374)	3.79	0.051
	T	25 (69.4%)	62 (57.4%)	1.686 (0.754-3.772)	1.64	0.200
	A	11 (30.6%)	46 (42.6%)			
	AA+AT vs. TT			1.082 (0.361-3.240)	0.02	0.888
	AA vs. AT+TT			10.882 (0.612-193.665)	4.8	0.028

rs2227508	AA	14 (77.8%)	36 (66.7%)	Ref		
	AT	4 (22.2%)	14 (25.9%)	1.361 (0.382-4.852)	0.23	0.633
	TT	0 (0%)	4 (7.4%)	3.757 (0.181-70.702)	1.51	0.218
	A	32 (88.9%)	86 (79.6%)	2.047 (0.654-6.399)	1.56	0.211
	T	4 (11.1%)	22 (20.4%)			
	TT+AT vs. AA			1.750 (0.503-6.089)	0.79	0.375
	TT vs. AT+AA			3.297 (0.169-64.262)	1.41	0.234

rs2227478	TT	11 (61.1%)	24 (44.4%)	Ref		
	CT	4 (22.2%)	20 (37%)	2.292 (0.631-8.317)	1.64	0.200
	CC	3 (16.7%)	10 (18.5%)	1.528 (0.350-6.674)	0.32	0.571
	T	26 (72.2%)	68 (63%)	1.529(0.669-3.498)	1.02	0.312
	C	10 (27.8%)	40 (37%)			
	CC+CT vs. TT			1.964 (0.661-5.837)	1.5	0.220
	CC vs. CT+TT			1.136 (0.276-4.688)	0.03	0.859

rs2227481	CC	13 (72.2%)	38 (70.4%)	Ref		
	CT	4 (22.2%)	13 (24.1%)	1.112 (0.307-4.021)	0.03	0.871
	TT	1 (5.6%)	3 (5.5%)	1.026 (0.098-10.752)	0	0.982
	C	30 (83.3%)	89 (82.4%)	1.067 (0.390-2.921)	0.02	0.898
	T	6 (16.7%)	19 (17.6%)			
	TT+CT vs. CC			1.095 (0.335-3.582)	0.02	0.881
	TT vs. CT+CC			1.00 (0.097-10.265)	0	1

rs2227491	AA	5 (27.7%)	20 (37%)	Ref		
	AG	12 (66.7%)	22 (40.7%)	0.458 (0.137-1.531)	1.64	0.199
	GG	1 (5.6%)	12 (22.2%)	3 (0.312-28.841)	0.97	0.336
	A	22 (61.1%)	62 (57.4%)	1.166 (0.539-2.521)	0.15	0.696
	G	14 (38.9%)	46 (42.6%)			
	GG+AG vs. AA			0.6654 (0.203-2.107)	0.51	0.474
	GG vs. AG+AA			4.857 (0.585-40.32)	2.53	0.111

rs2227483	TT	12 (66.7%)	46 (85.2%)	Ref		
	AT	1 (5.6%)	1 (1.9%)	0.261 (0.015-4.481)	0.98	0.322
	AA	5 (27.7%)	7 (12.9%)	0.365(0.098-1.356)	2.38	0.122
	T	25 (69.4%)	93 (86.1%)	0.367 (0.150-0.897)	5.07	0.024
	A	11 (30.6%)	15 (13.9%)			
	AA+AT vs. TT			0.348 (0.101-1.195)	2.96	0.085
	AA vs. AT+TT			0.387 (0.105-1.423)	2.13	0.144

**Table 6:** Haplotypes of IL-22 gene when control group was compared to patients' group.

Haplotype (block 1)			Freq.	Case, control ratio counts	Case, control frequencies	Chi square	*P* value
rs1179246	rs1182844	rs976748					
A	T	T	0.41	189.3 : 310.7, 179.3 : 220.7	0.379, 0.448	4.465	0.034
C	A	T	0.356	186.3 : 313.7, 134.3 : 265.7	0.373, 0.336	1.312	0.252
C	T	T	0.188	95.7 : 404.3, 73.7 : 326.3	0.191, 0.184	0.075	0.783
A	T	C	0.026	15.0 : 485.0, 8.0 : 392.0	0.030, 0.020	0.892	0.344
A	A	T	0.02	13.7 : 486.3, 4.7 : 395.3	0.027, 0.012	2.733	0.098

Haplotype (block 2)							
rs2046068	rs2227491						
A	G		0.494	231.9 : 268.1, 212.9 : 187.1	0.464, 0.532	4.174	0.041
C	A		0.306	159.9 : 340.1, 115.9 : 284.1	0.320, 0.290	0.94	0.332
A	A		0.198	107.1 : 392.9, 71.1 : 328.9	0.214, 0.178	1.866	0.171

**Table 7:** Haplotypes of IL-22 gene when group I+II of parasite density was compared to group III+IV.

Haplotypes						Freq.	Case, control ratio counts	Case, control frequencies	Chi square	*P* value
rs1179246	rs1182844	rs2046068	rs2227491	rs2227481	rs2227478					
A	T	A	G	C	T	0.374	158.1 : 265.9, 28.7 : 47.3	0.373, 0.378	0.007	0.933
C	A	A	A	C	T	0.145	63.9 : 360.1, 8.6 : 67.4	0.151, 0.113	0.732	0.392
C	A	C	A	C	C	0.137	52.3 : 371.7, 16.2 : 59.8	0.123, 0.213	4.386	0.036
C	T	C	A	T	C	0.1	44.8 : 379.2, 5.2 : 70.8	0.106, 0.069	0.959	0.327
C	A	A	A	C	C	0.038	16.9 : 407.1, 2.3 : 73.7	0.040, 0.030	0.178	0.672
C	A	C	A	C	T	0.032	15.9 : 408.1, 0.2 : 75.8	0.037, 0.003	2.457	0.117
C	T	C	A	C	C	0.017	6.6 : 417.4, 1.9 : 74.1	0.016, 0.025	0.323	0.569
C	T	A	G	C	T	0.017	6.8 : 417.2, 1.4 : 74.6	0.016, 0.019	0.024	0.877
A	T	A	G	C	C	0.016	6.9 : 417.1, 1.3 : 74.7	0.016, 0.017	0.003	0.955
C	T	A	G	C	C	0.015	7.4 : 416.6, 0.0 : 76.0	0.018, 0.000	1.312	0.252
C	T	A	G	T	C	0.014	7.1 : 416.9, 0.0 : 76.0	0.017, 0.000	1.292	0.255
C	T	A	G	C	C	0.013	4.5 : 419.5, 1.7 : 74.3	0.011, 0.023	0.778	0.377
A	A	C	A	C	C	0.012	5.8 : 418.2, 0.1 : 75.9	0.014, 0.001	0.869	0.351

**Table 8:** Haplotypes of IL-22 gene when group 1-5 years of age was compared to 6 years and above.

Haplotypes		Freq.	Case, control ratio counts	Case, control frequencies	Chi square	*P* value
rs2046068	rs2227491					
A	G	0.463	221.7 : 264.3, 10.0 : 4.0	0.456, 0.714	3.637	0.056
C	A	0.319	158.7 : 327.3, 1.0 : 13.0	0.327, 0.071	4.084	0.043
A	A	0.215	104.3 : 381.7, 3.0 : 11.0	0.215, 0.215	0	0.999

**Table 9:** Haplotypes of IL-22 gene when group 1-9 years of age was compared to 10 years and above.

Haplotype		Freq.	Case, control ratio counts	Case, control frequencies	Chi square	*P* value
rs2046068	rs2227491					
A	G	0.463	209.7 : 254.3, 22.0 : 14.0	0.452, 0.611	3.378	0.066
C	A	0.319	153.7 : 310.3, 6.0 : 30.0	0.331, 0.166	4.193	0.041
A	A	0.215	99.3 : 364.7, 8.0 : 28.0	0.214, 0.223	0.015	0.901

**Table 10:** Haplotypes of IL-22 gene when group 1-9 years of age was compared to 41 years and above.

Haplotype		Freq.	Case, control ratio counts	Case, control frequencies	Chi Square	*P* value
rs2046068	rs2227491					
A	G	0.472	46.0 : 62.0, 22.0 : 14.0	0.426, 0.611	3.715	0.053
C	A	0.299	37.0 : 71.0, 6.0 : 30.0	0.343, 0.167	3.99	0.045
A	A	0.229	25.0 : 83.0, 8.0 : 28.0	0.231, 0.222	0.013	0.908

## Data Availability

The data used to support the findings of this study are available from the corresponding author upon request.

## Supplementary Materials

Supplementary Materials Table S1: marker information of IL-22 SNP used in this study. Table S2: list of primer sequences used in this study for PCR and genotyping analysis of IL-22 SNPs. F: forward primer. R: reverse primer. Table S3: list of primer sequences used to generate IL-22 promoter constructs. Table S4: genotype distribution and allele frequency of IL-22 SNPs in group I of parasite density compared to group II+III+IV. Table S5: genotype distribution and allele frequency of IL-22 SNPs in group I+II of parasite density compared to group III+IV. Table S6: genotype distribution and allele frequency of IL-22 SNPs in group 10-20 years of age compared to 21 years and above age group.

## References

[1] Recker M., Bull P. C., Buckee C. O. (2018). Recent advances in the molecular epidemiology of clinical malaria. *F1000Research*.

[2] Trampuz A., Jereb M., Muzlovic I., Prabhu R. M. (2003). Clinical review: severe malaria. *Critical Care*.

[3] WHO (2018). *Malaria World Report 2017*.

[4] Al Zahrani M. H., Omar A. I., Abdoon A. M. O. (2018). Cross-border movement, economic development and malaria elimination in the Kingdom of Saudi Arabia. *BMC Medicine*.

[5] El Hassan I. M., Sahly A., Alzahrani M. H. (2015). Progress toward malaria elimination in Jazan Province, Kingdom of Saudi Arabia: 2000–2014. *Malaria Journal*.

[6] Soliman R. H., Garcia-Aranda P., Elzagawy S. M. (2018). Imported and autochthonous malaria in West Saudi Arabia: results from a reference hospital. *Malaria Journal*.

[7] de Mendonça V. R. R., Goncalves M. S., Barral-Netto M. (2012). The host genetic diversity in malaria infection. *Journal of Tropical Medicine*.

[8] Kwiatkowski D. P. (2005). How malaria has affected the human genome and what human genetics can teach us about malaria. *American Journal of Human Genetics*.

[9] Driss A., Hibbert J. M., Wilson N. O., Iqbal S. A., Adamkiewicz T. V., Stiles J. K. (2011). Genetic polymorphisms linked to susceptibility to malaria. *Malaria Journal*.

[10] McGuire W., Knight J. C., Hill A. V. S., Allsopp C. E. M., Greenwood B. M., Kwiatkowski D. (1999). Severe malarial anemia and cerebral malaria are associated with different tumor necrosis factor promoter alleles. *The Journal of Infectious Diseases*.

[11] Sinha S., Mishra S. K., Sharma S. (2008). Polymorphisms of *TNF*-enhancer and gene for Fc*γ*RIIa correlate with the severity of falciparum malaria in the ethnically diverse Indian population. *Malaria Journal*.

[12] Dudakov J. A., Hanash A. M., van den Brink M. R. M. (2015). Interleukin-22: immunobiology and pathology. *Annual Review of Immunology*.

[13] Kebir H., Kreymborg K., Ifergan I. (2007). Human T_H_17 lymphocytes promote blood-brain barrier disruption and central nervous system inflammation. *Nature Medicine*.

[14] Ouyang W. (2010). Distinct roles of IL-22 in human psoriasis and inflammatory bowel disease. *Cytokine & Growth Factor Reviews*.

[15] Li S., Yu M., Li H., Zhang H., Jiang Y. (2012). IL-17 and IL-22 in cerebrospinal fluid and plasma are elevated in Guillain-Barré syndrome. *Mediators of Inflammation*.

[16] Wang P., Bai F., Zenewicz L. A. (2012). IL-22 signaling contributes to West Nile encephalitis pathogenesis. *PLoS One*.

[17] Chi H. G., Zheng X. B., Wu Z. G., Dai S. X., Wan Z., Zou Y. (2014). Association of the interleukin-22 genetic polymorphisms with ulcerative colitis. *Diagnostic Pathology*.

[18] Saeki H., Hirota T., Nakagawa H. (2013). Genetic polymorphisms in the *IL22* gene are associated with psoriasis vulgaris in a Japanese population. *Journal of Dermatological Science*.

[19] Zhao T., Wu X., Liu J. (2015). Association between interleukin-22 genetic polymorphisms and bladder cancer risk. *Clinics (São Paulo, Brazil)*.

[20] Wang R., Zeng Y. L., Qin H. M. (2018). Association of interleukin 22 gene polymorphisms and serum IL-22 level with risk of systemic lupus erythematosus in a Chinese population. *Clinical & Experimental Immunology*.

[21] Koch O., Rockett K., Jallow M., Pinder M., Sisay-Joof F., Kwiatkowski D. (2005). Investigation of malaria susceptibility determinants in the *IFNG/IL26*/*IL22* genomic region. *Genes and Immunity*.

[22] Marquet S., Conte I., Poudiougou B. (2017). A functional *IL22* polymorphism (rs2227473) is associated with predisposition to childhood cerebral malaria. *Scientific Reports*.

[23] Mastelic B., do Rosario A. P. F., Veldhoen M. (2012). IL-22 protects against liver pathology and lethality of an experimental blood-stage malaria infection. *Frontiers in Immunology*.

[24] WHO (2010). *Basic Malaria Microscopy. 2*.

[25] Shaikh A. (2019). For the first time ever, a malaria vaccine is being rolled out in three African countries. Can it eliminate malaria for good?. https://www.undispatch.com/malaria-vaccine/.

[26] Bartoloni A., Zammarchi L. (2012). Clinical aspects of uncomplicated and severe malaria. *Mediterranean Journal of Hematology and Infectious Diseases*.

[27] Leoratti F. M. S., Farias L., Alves F. P. (2008). Variants in the toll-like receptor signaling pathway and clinical outcomes of malaria. *The Journal of Infectious Diseases*.

[28] Oyegue-Liabagui S. L., Bouopda-Tuedom A. G., Kouna L. C. (2017). Pro- and anti-inflammatory cytokines in children with malaria in Franceville, Gabon. *American Journal of Clinical and Experimental Immunology*.

[29] Ryan-Payseur B., Ali Z., Huang D. (2011). Virus infection stages and distinct Th1 or Th17/Th22 T-cell responses in malaria/SHIV coinfection correlate with different outcomes of disease. *The Journal of Infectious Diseases*.

[30] Zhang Y., Li J., Wang C., Zhang L. (2015). Association between the interaction of key genes involved in effector T-cell pathways and susceptibility to develop allergic rhinitis: a population-based case-control association study. *PLoS One*.

[31] Nikamo P., Cheuk S., Lysell J. (2014). Genetic variants of the *IL22* promoter associate to onset of psoriasis before puberty and increased IL-22 production in T cells. *The Journal of Investigative Dermatology*.

[32] Zhang G., Chen X., Chan L. (2011). An SNP selection strategy identified IL-22 associating with susceptibility to tuberculosis in Chinese. *Scientific Reports*.

[33] Gonçalves B. P., Huang C. Y., Morrison R. (2014). Parasite burden and severity of malaria in Tanzanian children. *The New England Journal of Medicine*.

[34] Mangal P., Mittal S., Kachhawa K., Agrawal D., Rath B., Kumar S. (2017). Analysis of the clinical profile in patients with *Plasmodium falciparum* malaria and its association with parasite density. *Journal of Global Infectious Diseases*.

[35] Weger W., Hofer A., Wolf P. (2009). Common polymorphisms in the interleukin-22 gene are not associated with chronic plaque psoriasis. *Experimental Dermatology*.

[36] Song R. H., Li Q., Wang W., Yao Q. M., Shao X. Q., Zhang J. A. (2017). Variants of Interleukin-22 gene confer predisposition to autoimmune thyroid disease. *International Journal of Endocrinology*.

[37] Jacquier A. (1996). Group II introns: elaborate ribozymes. *Biochimie*.

[38] Qaddourah R. H., Magdoud K., Saldanha F. L. (2014). IL-10 gene promoter and intron polymorphisms and changes in IL-10 secretion in women with idiopathic recurrent miscarriage. *Human Reproduction*.

[39] Hu J., Li Y., Chen L. (2016). Impact of *IL-22* gene polymorphism on human immunodeficiency virus infection in Han Chinese patients. *Journal of Microbiology, Immunology, and Infection*.

