# Supplementary material for: Interleukin-22 Polymorphisms in Plasmodium falciparum-Infected Malaria Patients
Source: Mediators Inflamm. 2020 Feb 19;2020:5193723. doi: 10.1155/2020/5193723 (PMC7049855; doi:10.1155/2020/5193723)
Supplement: Supplementary Materials — Table S1: marker information of IL-22 SNP used in this study. Table S2: list of primer sequences used in this study for PCR and genotyping analysis of IL-22 SNPs. F: forward primer. R: reverse primer. Table S3: list of primer sequences used to generate IL-22 promoter constructs. Table S4: genotype distribution and allele frequency of IL-22 SNPs in group I of parasite density compared to group II+III+IV. Table S5: genotype distribution and allele frequency of IL-22 SNPs in group I+II of parasite density compared to group III+IV. Table S6: genotype distribution and allele frequency of IL-22 SNPs in group 10-20 years of age compared to 21 years and above age group. [file 5193723.f1.docx]

| **Table S1:** Marker information of IL-22 SNP used in this study. | | | | | | |
| --- | --- | --- | --- | --- | --- | --- |
| Name | Position | ObsHET | PredHET | HWpval | MAF | Alleles |
| rs1179246 | 68640583 | 0.467 | 0.496 | 0.235 | 0.456 | C:A |
| rs1182844 | 68641532 | 0.478 | 0.47 | 0.806 | 0.377 | T:A |
| rs2227508 | 68641928 | 0.282 | 0.294 | 0.477 | 0.179 | A:T |
| rs976748 | 68643594 | 0.042 | 0.05 | 0.058 | 0.026 | T:C |
| rs2046068 | 68645975 | 0.416 | 0.426 | 0.658 | 0.308 | A:C |
| rs2227491 | 68646521 | 0.493 | 0.5 | 0.833 | 0.496 | A:G |
| rs2227481 | 68648341 | 0.28 | 0.309 | 0.066 | 0.191 | C:T |
| rs2227478 | 68648622 | 0.438 | 0.467 | 0.206 | 0.372 | T:C |

ObsHet: Observed heterozygous alleles; PredHET: Predicted heterozygous alleles; HWpval: Hardy-Weinberg equilibrium p value; MAF: Minor allele frequency.

| **Table S2:** List of primer sequences used in this study for PCR and genotyping analysis of IL-22 SNPs. F: Forward primer. R: Reverse primer. | | |
| --- | --- | --- |
| **SNPs** | **Primer sequence 5' -> 3'** | **Amplification conditions** |
| rs1179246 | F:AACAGAATGGCTGGCTGAGT  R:GTGGGCTGAAAGGTGTCC | 95 °C 2 min (1X), 95 °C 30 sec, **60 °C** 30 sec,72 °C 48 sec (40X), 72 C 5min(1X) |
| rs2046068 | F:TCAGGAGTCATTTGGGATCA R:AATGTTCAGCGGCATCAAGT |  |
| rs2227491 | F:ATGATGTTGCTCTGGGGAGA R:GCACCACCTCCTGCATATAA |  |
| rs1182844 | F:CTGGCATCCTTTGTCCATTC R:AAAAGAAAGGAATGATGTTGCTG |  |
| rs976748 | F:AAAATGCATGACAGACAGTAGGG R:CCTAACATTCATTCTTTCTCTCTCC | 95 C 2 min (1X), 95 °C 30 sec, **55 °C** 30 sec,72 °C48 sec (40X), 72 °C 5min(1X) |
| rs2227481 | F:GCCACCTTCACAAATGCTCA R:ACACTTCACACAAGTCTCAACA |  |
| rs2227478 | F:GCCACCTTCACAAATGCTCAG  R: GAGGTTCCGCGTTACCTTCAA | 95 °C 2 min (1X), 95 °C 30 sec,**65 °C** 30 sec, 72 °C 48 sec (40X), 72 °C 5min(1X) |
| rs2227508 | F:TCATTCGATATTGCTACTTGAGTG R:TTTTCTTTTCAAAGGTACATGCTG | 95 C 2 min (1X), 95 C 1 min, **61 C** 30 sec, 72 °C 60 sec (40X), 72 °C 5min(1X) |
| rs2227483 | F:TCCCTTCTTCAGACATCCATG R:TGACAGCTAACAGATCATTCCA | 95 C 2 min (1X), 95 °C 30 sec, **60 °**C 30 sec, 72 °C 48 sec (40X), 72 °C 5min(1X) |

| **Table S3:** List of primer sequences used to generate IL-22 promoter constructs. | | | | | |
| --- | --- | --- | --- | --- | --- |
| **Construct Name** | **Annealing Temp.** | Chromosomal location* | | **Primer sequence 5'->3'** | Size (bp) |
|  |  | Start | End |  |  |
| F1R2 | 62^O^C | 68,647,573 | 68,648,430 | F:CGCCTACCCGGGATTTGCTTCCTTGCTCTGCG  R: TAGGCG**AGATCT**ACGTCACTATTAGAGCCCGG | 857 |
| F2R1 | 63^O^C | 68,647,242 | 68,647,857 | F:CGCCTACCCGGGTGGAATGATCTGTTAGCTGTCA R:TAGGCG**AGATCT**AACTCGAGCAACTGGTGACT | 615 |
| F2R2 | 62^O^C | 68,647,573 | 68,647,857 | F:CGCCTACCCGGGTGGAATGATCTGTTAGCTGTCA  R:TAGGCG**AGATCT**ACGTCACTATTAGAGCCCGG | 284 |
| F3R1 | 60^O^C | 68,647,242 | 68,647,737 | F:CGCCTACCCGGGTCCGTGACCAAAATGCTTACTC  R: TAGGCG**AGATCT**AACTCGAGCAACTGGTGACT | 495 |
| F3R2 | 60^O^C | 68,647,573 | 68,647,737 | F:CGCCTACCCGGGTCCGTGACCAAAATGCTTACTC R:TAGGCG**AGATCT**ACGTCACTATTAGAGCCCGG | 164 |

F: Forward

R: Reverse

* Homo sapiens:GRCh37.p13 (GCF_000001405.25)Chr 12 (NC_000012.11).

SmaI restriction recognition sequence is underlined.

BglII restriction recognition sequence is in bold.

| **Table S4:** Genotype distribution and allele frequency of IL-22 SNPs in group I of parasite density compared to group II+III+IV. | | | | | | |
| --- | --- | --- | --- | --- | --- | --- |
| **SNPs** | **Genotype/ Allele distribution** | **Group-I** | **Group-** **II+III+IV** | **OR (95% C.I.)** | **χ²** | **P-value** |
|  |  |  |  |  |  |  |
| **rs976748** | **TT** | **13 (92.9%)** | **223 (94.5%)** | **Ref** |  |  |
|  | **CT** | **1 (7.1%)** | **12 (5.1%)** | **0.700(0.084-5.800)** | **0.11** | **0.739** |
|  | **CC** | **0 (0%)** | **1 (0.4%)** | **0.181 (0.007-4.661)** | **0.06** | **0.809** |
|  | **T** | **27 (96.4%)** | **458 (97%)** | **0.825 (0.105-6.512)** | **0.03** | **0.584** |
|  | **C** | **1 (3.6%)** | **14 (3%)** |  |  |  |
|  | **CC+CT vs TT** |  |  | **0.758 (0.092-6.247)** | **0.07** | **0.796** |
|  | **CC vs CT+TT** |  |  | **5.414 (0.211-138.822)** | **0.06** | **0.807** |
| **rs1179246** | **CC** | **5 (35.7%)** | **81 (34.3%)** | **Ref** |  |  |
|  | **AC** | **5 (35.7%)** | **107 (45.3%)** | **1.321 (0.370-4.717)** | **0.18** | **0.667** |
|  | **AA** | **4 (28.6%)** | **48 (20.3%)** | **0.741 (0.190-2.893)** | **0.19** | **0.664** |
|  | **C** | **15 (53.6%)** | **269 (57%)** | **0.871 (0.405-1.871)** | **0.13** | **0.722** |
|  | **A** | **13 (46.4%)** | **203 (43%)** |  |  |  |
|  | **AA+AC vs CC** |  |  | **1.1063 (0.345-3.277)** | **0.01** | **0.915** |
|  | **AA vs AC+CC** |  |  | **1.567 (0.471-5.212)** | **0.54** | **0.460** |
| **rs2046068** | **AA** | **8 (57.1%)** | **106 (44.9%)** | **Ref** |  |  |
|  | **AC** | **6 (42.9%)** | **105 (44.5%)** | **1.321 (0.443-3.938)** | **0.25** | **0.616** |
|  | **CC** | **0 (0%)** | **25 (10.6%)** | **4.070 (0.227-72.854)** | **1.86** | **0.172** |
|  | **A** | **22 (78.6%)** | **317 (67.2%)** | **1.793 (0.712-4.512)** | **1.58** | **0.209** |
|  | **C** | **6 (21.4%)** | **155 (32.8%)** |  |  |  |
|  | **CC+AC vs AA** |  |  | **1.635 (0.550-4.859)** | **0.8** | **0.372** |
|  | **CC vs AC+AA** |  |  | **0.286 (0.017-4.939)** | **1.65** | **0.199** |
| **rs1182844** | **TT** | **6 (42.9%)** | **84 (35.6%)** | **Ref** |  |  |
|  | **AT** | **5 (35.7%)** | **115 (48.7%)** | **1.643 (0.485-5.563)** | **0.65** | **0.420** |
|  | **AA** | **3 (21.4%)** | **37 (15.7%)** | **0.881 (0.209-3.714)** | **0.03** | **0.862** |
|  | **T** | **17 (60.7%)** | **283 (60%)** | **1.032 (0.473-2.253)** | **0.01** | **0.936** |
|  | **A** | **11 (39.3%)** | **189 (40%)** |  |  |  |
|  | **AA+AT vs TT** |  |  | **1.357 (0.456-4.043)** | **0.3** | **0.582** |
|  | **AA vs AT+TT** |  |  | **1.467 (0.390-5.513)** | **0.33** | **0.568** |
| **rs2227508** | **AA** | **10 (71.4%)** | **156 (66.1%)** | **Ref** |  |  |
|  | **AT** | **4 (28.6%)** | **67 (28.4%)** | **1.074 (0.325-3.545)** | **0.01** | **0.907** |
|  | **TT** | **0 (0%)** | **13 (5.5%)** | **1.812 (0.101-32.627)** | **0.83** | **0.362** |
|  | **A** | **24 (85.7%)** | **379 (80.3%)** | **1.472 (0.499-4.346)** | **0.5** | **0.481** |
|  | **T** | **4 (14.3%)** | **93 (19.7%)** |  |  |  |
|  | **TT+AT vs AA** |  |  | **1.282 (0.390-4.216)** | **0.17** | **0.681** |
|  | **TT vs AT+AA** |  |  | **0.571 (0.032-10.091)** | **0.81** | **0.367** |
| **rs2227478** | **TT** | **5 (35.7%)** | **95 (40.3%)** | **Ref** |  |  |
|  | **CT** | **8 (57.1%)** | **107 (45.3%)** | **0.704 (0.223-2.225)** | **0.36** | **0.548** |
|  | **CC** | **1 (7.1%)** | **34 (14.4%)** | **1.789 (0.202-15.869)** | **0.28** | **0.596** |
|  | **T** | **18 (64.3%)** | **297 (62.9%)** | **1.061 (0.479-2.349)** | **0.02** | **0.884** |
|  | **C** | **10 (35.7%)** | **175 (37.1%)** |  |  |  |
|  | **CC+CT vs TT** |  |  | **0.825 (0.268-2.537)** | **0.11** | **0.736** |
|  | **CC vs CT+TT** |  |  | **0.457 (0.05-3.608)** | **0.58** | **0.446** |
| **rs2227481** | **CC** | **12 (85.7%)** | **163 (69.1%)** | **Ref** |  |  |
|  | **CT** | **2 (14.3%)** | **67 (28.4%)** | **2.466 (0.537-11.318)** | **1.43** | **0.231** |
|  | **TT** | **0 (0%)** | **6 (2.5%)** | **0.994 (0.053-18.676)** | **0.44** | **0.506** |
|  | **C** | **26 (92.9%)** | **393 (83.3%)** | **2.613(0.608-11.234)** | **1.79** | **0.287** |
|  | **T** | **2 (7.1%)** | **79 (16.7%)** |  |  |  |
|  | **TT+CT vs CC** |  |  | **2.687 (0.586-12.313)** | **1.74** | **0.186** |
|  | **TT vs CT+CC** |  |  | **1.223 (0.066-22.786)** | **0.36** | **0.545** |
| **rs2227491** | **AA** | **5 (35.7%)** | **67 (28.4%)** | **Ref** |  |  |
|  | **AG** | **6 (42.9%)** | **117 (49.6%)** | **1.455 (0.428-4.950)** | **0.36** | **0.546** |
|  | **GG** | **3 (21.4%)** | **52 (22%)** | **1.294 (0.295-5.662)** | **0.12** | **0.732** |
|  | **A** | **16 (57.1%)** | **251 (53.2%)** | **1.174 (0.544-2.536)** | **0.17** | **0.682** |
|  | **G** | **12 (42.9%)** | **221 (46.8%)** |  |  |  |
|  | **GG+AG vs AA** |  |  | **1.401 (0.453-4.335)** | **0.35** | **0.556** |
|  | **GG vs AG+AA** |  |  | **0.965 (0.260-3.588)** | **0** | **0.957** |
| **rs2227483** | **TT** | **11 (78.6%)** | **193 (81.8%)** | **Ref** |  |  |
|  | **AT** | **0 (0%)** | **13 (5.5%)** | **1.605 (0.090-28.720)** | **0.74** | **0.390** |
|  | **AA** | **3 (21.4%)** | **30 (12.7%)** | **0.570 (0.150-2.162)** | **0.7** | **0.403** |
|  | **T** | **22 (78.6%)** | **399 (84.5%)** | **0.671 (0.263-1.711)** | **0.71** | **0.421** |
|  | **A** | **6 (21.4%)** | **73 (15.5%)** |  |  |  |
|  | **AA+AT vs TT** |  |  | **0.817 (0.219-3.054)** | **0.09** | **0.763** |
|  | **AA vs AT+TT** |  |  | **1.873 (0.494-7.101)** | **0.88** | **0.349** |
|  |  |  |  |  |  |  |

| **Table S5:** Genotype distribution and allele frequency of IL-22 SNPs in group I+II of parasite density compared to group III+IV. | | | | | | |
| --- | --- | --- | --- | --- | --- | --- |
| **SNPs** | **Genotype/ Allele distribution** | **Group-** **I+II** | **Group-** **III+IV** | **OR (95% C.I.)** | **χ²** | **P-value** |
|  |  |  |  |  |  |  |
| **rs976748** | **TT** | **37 (97.4%)** | **199 (93.9%)** | **Ref** |  |  |
|  | **CT** | **1 (2.6%)** | **12 (5.7%)** | **2.231 (0.282-17.681)** | **0.61** | **0.435** |
|  | **CC** | **0 (0%)** | **1 (0.4%)** | **0.564 (0.023-14.107)** | **0.19** | **0.666** |
|  | **T** | **75 (98.7%)** | **410 (96.7%)** | **2.561 (0.332-19.767)** | **0.87** | **0.711** |
|  | **C** | **1 (1.3%)** | **14 (3.3%)** |  |  |  |
|  | **CC+CT vs TT** |  |  | **2.417 (0.307-19.040)** | **0.75** | **0.387** |
|  | **CC vs CT+TT** |  |  | **1.831 (0.073-45.784)** | **0.18** | **0.671** |
| **rs1179246** | **CC** | **11 (28.9%)** | **74 (34.9%)** | **Ref** |  |  |
|  | **AC** | **19 (50%)** | **93 (43.9%)** | **0.728 (0.326-1.624)** | **0.61** | **0.436** |
|  | **AA** | **8 (21.1%)** | **45 (21.2%)** | **0.836 (0.313-2.235)** | **0.13** | **0.721** |
|  | **C** | **41 (54%)** | **241 (56.8%)** | **0.890 (0.545-1.452)** | **0.22** | **0.639** |
|  | **A** | **35 (46%)** | **183 (43.2%)** |  |  |  |
|  | **AA+AC vs CC** |  |  | **0.760 (0.357-1.618)** | **0.51** | **0.475** |
|  | **AA vs AC+CC** |  |  | **0.990 (0.424-2.307)** | **0** | **0.980** |
| **rs2046068** | **AA** | **17 (44.7%)** | **97 (45.7%)** | **Ref** |  |  |
|  | **AC** | **18 (47.4%)** | **93 (43.9%)** | **0.905 (0.440-1.863)** | **0.07** | **0.787** |
|  | **CC** | **3 (7.9%)** | **22 (10.4%)** | **1.285 (0.346-4.772)** | **0.14** | **0.707** |
|  | **A** | **52 (68.4%)** | **287 (67.7%)** | **1.034 (0.612-1.748)** | **0.02** | **0.899** |
|  | **C** | **24 (31.6%)** | **137 (32.3%)** |  |  |  |
|  | **CC+AC vs AA** |  |  | **0.960 (0.479-1.921)** | **0.01** | **0.907** |
|  | **CC vs AC+AA** |  |  | **0.740 (0.210-2.607)** | **0.22** | **0.638** |
| **rs1182844** | **TT** | **13 (34.2%)** | **77 (36.3%)** | **Ref** |  |  |
|  | **AT** | **18 (47.4%)** | **102 (48.1%)** | **0.957 (0.442-2.071)** | **0.01** | **0.910** |
|  | **AA** | **7 (18.4%)** | **33 (15.6%)** | **0.796(0.291-2.175)** | **0.2** | **0.655** |
|  | **T** | **44 (57.9%)** | **256 (60.4%)** | **0.902 (0.550-1.481)** | **0.17** | **0.684** |
|  | **A** | **32 (42.1%)** | **168 (39.6%)** |  |  |  |
|  | **AA+AT vs TT** |  |  | **0.912 (0.441-1.885)** | **0.06** | **0.802** |
|  | **AA vs AT+TT** |  |  | **1.225 (0.498-3.014)** | **0.2** | **0.658** |
| **rs2227508** | **AA** | **22 (57.9%)** | **144 (68%)** | **Ref** |  |  |
|  | **AT** | **13 (34.2%)** | **58 (27.3%)** | **0.682 (0.322-1.443)** | **1.01** | **0.314** |
|  | **TT** | **3 (7.9%)** | **10 (4.7%)** | **0.509 (0.130-1.996)** | **0.97** | **0.325** |
|  | **A** | **57 (75%)** | **346 (81.6%)** | **0.676 (0.381-1.201)** | **1.8** | **0.180** |
|  | **T** | **19 (25%)** | **78 (18.4%)** |  |  |  |
|  | **TT+AT vs AA** |  |  | **0.649 (0.321-1.315)** | **1.45** | **0.228** |
|  | **TT vs AT+AA** |  |  | **1.731 (0.454-6.607)** | **0.66** | **0.416** |
| **rs2227478** | **TT** | **13 (34.2%)** | **87 (32.1%)** | **Ref** |  |  |
|  | **CT** | **18 (47.4%)** | **97 (35.8%)** | **0.805 (0.373-1.739)** | **0.3** | **0.580** |
|  | **CC** | **7 (18.4%)** | **87 (32.1%)** | **0.598 (0.217-1.646)** | **1.01** | **0.315** |
|  | **T** | **44 (57.9%)** | **271 (50%)** | **0.776 (0.472-1.276)** | **1** | **0.316** |
|  | **C** | **32 (42.1%)** | **271 (50%)** |  |  |  |
|  | **CC+CT vs TT** |  |  | **0.747 (0.362-1.541)** | **0.63** | **0.428** |
|  | **CC vs CT+TT** |  |  | **1.484 (0.596-3.692)** | **0.73** | **0.393** |
| **rs2227481** | **CC** | **31 (81.6%)** | **144 (68%)** | **Ref** |  |  |
|  | **CT** | **7 (18.4%)** | **62 (29.2%)** | **1.907 (0.797-4.563)** | **2.16** | **0.141** |
|  | **TT** | **0 (0%)** | **6 (2.8%)** | **2.834 (0.156-51.614)** | **1.28** | **0.257** |
|  | **C** | **69 (90.8%)** | **350 (82.5%)** | **2.084 (0.921-4.717)** | **3.23** | **0.072** |
|  | **T** | **7 (9.2%)** | **74 (17.5%)** |  |  |  |
|  | **TT+CT vs CC** |  |  | **2.091 (0.877-4.989)** | **2.86** | **0.090** |
|  | **TT vs CT+CC** |  |  | **0.413 (0.023-7.476)** | **1.1** | **0.293** |
| **rs2227491** | **AA** | **9 (23.7%)** | **63 (29.7%)** | **Ref** |  |  |
|  | **AG** | **22 (57.9%)** | **101 (47.6%)** | **0.656 (0.284-1.515)** | **0.99** | **0.320** |
|  | **GG** | **7 (18.4%)** | **48 (22.6%)** | **0.980 (0.341-2.818)** | **0** | **0.969** |
|  | **A** | **40 (52.6%)** | **227 (53.5%)** | **0.964 (0.591-1.572)** | **0.02** | **0.884** |
|  | **G** | **36 (47.4%)** | **197 (46.5%)** |  |  |  |
|  | **GG+AG vs AA** |  |  | **0.734 (0.329-1.640)** | **0.57** | **0.449** |
|  | **GG vs AG+AA** |  |  | **0.772 (0.320-1.862)** | **0.33** | **0.563** |
| **rs2227483** | **TT** | **30 (78.9%)** | **174 (82.1%)** | **Ref** |  |  |
|  | **AT** | **2 (5.3%)** | **11 (5.2%)** | **0.948(0.200-4.493)** | **0** | **0.946** |
|  | **AA** | **6 (15.8%)** | **27 (12.7%)** | **0.776 (0.295-2.038)** | **0.27** | **0.605** |
|  | **T** | **62 (81.6%)** | **359 (84.7%)** | **0.802 (0.424-1.517)** | **0.46** | **0.496** |
|  | **A** | **14 (18.4%)** | **65 (15.3%)** |  |  |  |
|  | **AA+AT vs TT** |  |  | **0.819 (0.348-1.926)** | **0.21** | **0.646** |
|  | **AA vs AT+TT** |  |  | **1.285 (0.491-3.358)** | **0.26** | **0.608** |
|  |  |  |  |  |  |  |

| **Table S6:** Genotype distribution and allele frequency of IL-22 SNPs in group 10-20 years of age compared to 21 years and above age group. | | | | | | |
| --- | --- | --- | --- | --- | --- | --- |
| **SNPs** | **Genotype/ Allele distribution** | **10-20years** | **21 years and above** | **OR (95% C.I.)** | **χ²** | **P-value** |
|  |  |  |  |  |  |  |
| **rs976748** | **TT** | **51 (94.4%)** | **169 (95.5%)** | **Ref** |  |  |
|  | **CT** | **2 (3.7%)** | **8 (4.5%)** | **1.207 (0.248-5.865)** | **0.05** | **0.815** |
|  | **CC** | **1 (1.9%)** | **0 (0%)** | **0.101(0.004-2.524)** | **3.26** | **0.070** |
|  | **T** | **104 (96.3%)** | **346 (97.7%)** | **0.601 (0.177-2.036)** | **0.68** | **0.487** |
|  | **C** | **4 (3.7%)** | **8 (2.3%)** |  |  |  |
|  | **CC+CT vs TT** |  |  | **0.805 (0.206-3.146)** | **0.1** | **0.754** |
|  | **CC vs CT+TT** |  |  | **9.953 (0.400-247.912)** | **3.29** | **0.069** |
| **rs1179246** | **CC** | **18 (33.3%)** | **62 (35%)** | **Ref** |  |  |
|  | **AC** | **23 (42.6%)** | **81 (45.8%)** | **1.347 (0.612-2.965)** | **0.55** | **0.459** |
|  | **AA** | **13 (24.1%)** | **34 (19.2%)** | **1.317 (0.576-3.011)** | **0.43** | **0.513** |
|  | **C** | **59 (54.6%)** | **205 (58%)** | **1.143 (0.741-1.763)** | **0.36** | **0.546** |
|  | **A** | **49 (45.4%)** | **149 (42%)** |  |  |  |
|  | **AA+AC vs CC** |  |  | **1.334 (0.644-2.760)** | **0.6** | **0.436** |
|  | **AA vs AC+CC** |  |  | **0.927 (0.487-1.767)** | **0.05** | **0.818** |
| **rs2046068** | **AA** | **21 (38.9%)** | **80 (45.2%)** | **Ref** |  |  |
|  | **AC** | **25 (46.3%)** | **80 (45.2%)** | **0.840(0.435-1.622)** | **0.27** | **0.603** |
|  | **CC** | **8 (14.8%)** | **17 (9.6%)** | **0.558 (0.212-1.469)** | **1.42** | **0.233** |
|  | **A** | **67 (62%)** | **240 (67.8%)** | **0.776 (0.496-1.215)** | **1.23** | **0.267** |
|  | **C** | **41 (38%)** | **114 (32.2%)** |  |  |  |
|  | **CC+AC vs AA** |  |  | **0.772 (0.414-1.437)** | **0.67** | **0.413** |
|  | **CC vs AC+AA** |  |  | **1.637 (0.664-4.034)** | **1.16** | **0.280** |
| **rs1182844** | **TT** | **17 (31.5%)** | **65 (36.7%)** | **Ref** |  |  |
|  | **AT** | **28 (51.8%)** | **81 (45.8%)** | **0.757 (0.381-1.501)** | **0.64** | **0.424** |
|  | **AA** | **9 (16.7%)** | **31 (17.5%)** | **0.901 (0.361-2.247)** | **0.05** | **0.822** |
|  | **T** | **62 (57.4%)** | **211 (59.6%)** | **0.913 (0.590-1.413)** | **0.17** | **0.684** |
|  | **A** | **46 (42.6%)** | **143 (40.4%)** |  |  |  |
|  | **AA+AT vs TT** |  |  | **0.792 (0.413-1.517)** | **0.5** | **0.481** |
|  | **AA vs AT+TT** |  |  | **0.942 (0.417-2.126)** | **0.02** | **0.885** |
| **rs2227508** | **AA** | **33 (61.1%)** | **118 (66.7%)** | **Ref** |  |  |
|  | **AT** | **16 (29.6%)** | **51 (28.8%)** | **0.891 (0.451-1.762)** | **0.11** | **0.740** |
|  | **TT** | **5 (9.3%)** | **8 (4.5%)** | **0.447 (0.137-1.459)** | **1.85** | **0.173** |
|  | **A** | **82 (76%)** | **287 (81%)** | **0.736 (0.440-1.232)** | **1.36** | **0.242** |
|  | **T** | **26 (24%)** | **67 (19%)** |  |  |  |
|  | **TT+AT vs AA** |  |  | **0.786(0.418-1.475)** | **0.56** | **0.452** |
|  | **TT vs AT+AA** |  |  | **2.156 (0.675-6.888)** | **1.75** | **0.185** |
| **rs2227478** | **TT** | **16 (29.6%)** | **72 (40.7%)** | **Ref** |  |  |
|  | **CT** | **28 (51.9%)** | **83 (46.9%)** | **0.659 (0.330-1.314)** | **1.41** | **0.234** |
|  | **CC** | **10 (18.5%)** | **22 (12.4%)** | **0.489 (0.19-1.231)** | **2.36** | **0.124** |
|  | **T** | **60 (55.6%)** | **227 (64.1%)** | **0.699 (0.452-1.083)** | **2.58** | **0.108** |
|  | **C** | **48 (44.4%)** | **127 (35.9%)** |  |  |  |
|  | **CC+CT vs TT** |  |  | **0.614 (0.318-1.184)** | **2.14** | **0.143** |
|  | **CC vs CT+TT** |  |  | **1.601 (0.706-3.632)** | **1.29** | **0.256** |
| **rs2227481** | **CC** | **37 (68.5%)** | **124 (70%)** | **Ref** |  |  |
|  | **CT** | **16 (29.6%)** | **49 (27.7%)** | **0.914 (0.466-1.792)** | **0.07** | **0.792** |
|  | **TT** | **1 (1.9%)** | **4 (2.3%)** | **1.194 (0.129-11.010)** | **0.02** | **0.875** |
|  | **C** | **90 (83.3%)** | **297 (83.9%)** | **0.960 (0.537-1.714)** | **0.02** | **0.889** |
|  | **T** | **18 (16.7%)** | **57 (16.1%)** |  |  |  |
|  | **TT+CT vs CC** |  |  | **0.930 (0.482-1.797)** | **0.05** | **0.829** |
|  | **TT vs CT+CC** |  |  | **0.816 (0.089-7.460)** | **0.03** | **0.856** |
| **rs2227491** | **AA** | **18 (33.3%)** | **53 (30%)** | **Ref** |  |  |
|  | **AG** | **26 (48.2%)** | **85 (48%)** | **1.110 (0.556-2.218)** | **0.09** | **0.766** |
|  | **GG** | **10 (18.5%)** | **39 (22%)** | **1.325 (0.551-3.183)** | **0.4** | **0.529** |
|  | **A** | **62 (57.4%)** | **191 (54%)** | **1.150 (0.745-1.777)** | **0.4** | **0.528** |
|  | **G** | **46 (42.6%)** | **163 (46%)** |  |  |  |
|  | **GG+AG vs AA** |  |  | **1.170 (0.610-2.243)** | **0.22** | **0.636** |
|  | **GG vs AG+AA** |  |  | **0.804 (0.371-1.742)** | **0.31** | **0.580** |
| **rs2227483** | **TT** | **44 (81.5%)** | **148 (83.7%)** | **Ref** |  |  |
|  | **AT** | **3 (5.5%)** | **9 (5%)** | **0.892 (0.231-3.438)** | **0.03** | **0.867** |
|  | **AA** | **7 (13%)** | **20 (11.3%)** | **0.849(0.337-2.140)** | **0.12** | **0.729** |
|  | **T** | **91 (84.3%)** | **305 (86.2%)** | **0.860 (0.472-1.566)** | **0.24** | **0.621** |
|  | **A** | **17 (15.7%)** | **49 (13.8%)** |  |  |  |
|  | **AA+AT vs TT** |  |  | **0.862 (0.390-1.907)** | **0.13** | **0.713** |
|  | **AA vs AT+TT** |  |  | **1.169 (0.466-2.935)** | **0.11** | **0.739** |
|  |  |  |  |  |  |  |
